# A Quantitative Real-Time PCR Assay for Detection and Quantification of the Ginseng Alternaria Leaf and Stem Blight Pathogen *Alternaria panax*

**DOI:** 10.3390/jof12050317

**Published:** 2026-04-26

**Authors:** Jinling Lan, Yingxue Du, Mingxuan Xiong, Kaixin Zhang, Xiaolin Chen, Ying Song, Yuejia Song, Baohui Lu, Changqing Chen, Ronglin He, Jie Gao

**Affiliations:** 1 College of Plant Protection, Jilin Agricultural University, Changchun 130118, China; lanjl2017@163.com (J.L.); duyingxue@aliyun.com (Y.D.); mnb19982733209@163.com (M.X.); songying468@163.com (Y.S.); 18243472172@163.com (Y.S.); lbh860110@126.com (B.L.); chenchangqing@jlau.edu.cn (C.C.); 2 Jilin Provincial Ginseng and Deer Antler Department (Jilin Provincial Traditional Chinese Medicine Material Department Center), Changchun 130033, China; jlzhangkx@163.com (K.Z.); jlsrb@163.com (X.C.); 3 State-Local Joint Engineering Research Center of Ginseng Breeding and Application, Changchun 130118, China

**Keywords:** ginseng, phytopathogenic fungi, molecular detection, early diagnosis, seed, soil

## Abstract

Ginseng Alternaria leaf and stem blight, caused by *Alternaria panax*, imposes substantial yield and economic losses to the ginseng cultivation industry. Current diagnostic methods for ginseng diseases primarily rely on pathogen isolation from infected tissues, a procedure that is laborious, time-consuming, and inherently low in sensitivity. This study has therefore developed a rapid, specific and sensitive SYBR Green-based quantitative real-time PCR (qPCR) assay for detecting *A. panax* in plants, seeds, and soil. The developed qPCR assay exhibited high sensitivity and repeatability, with a detection limit of 0.074 fg/μL of target amplicon DNA (0.619 ng/μL of genomic DNA) and a coefficient of variation below 2%. In artificially inoculated tissues (leaves, stems and seeds), Ct values decreased progressively with increasing incubation time, reflecting pathogen proliferation. Analysis of field-collected leaves and stems showed a strong overall correlation between Ct values and visual disease grades. Surveying of ginseng-growing areas revealed that *A. panax* was detected in asymptomatic leaves and stems at rates of 12.12% and 14.29%, respectively, and in 14.46% of soil samples and 23.73% of seed samples. This qPCR assay presented here provides a robust tool for forecasting early disease, tracking the primary inoculum of the pathogen and its transmission chains, and screening of both ginseng seed lots and candidate soils for ginseng Alternaria leaf and stem blight prior to planting.

## 1. Introduction

Ginseng (*Panax ginseng* Meyer) is a well-known perennial medicinal plant that has been cultivated in China for over 1600 years [1]. The country contributes approximately 70% of the world’s total annual yield. However, the expansion of ginseng cultivation and the prolongation of growing periods have made it increasingly susceptible to fungal pathogens, leading to various diseases [2]. Ginseng Alternaria leaf and stem blight, mainly caused by *Alternaria panax*, occurs widely in ginseng-growing areas [3]. The fungal pathogens infect various parts of the plant, including roots, stems, leaves, and fruits. The annual incidence rate of ginseng Alternaria leaf and stem blight is 20–30%, but can reach up to 70% in severe years [4].

*A. panax* primarily overwinters as mycelium or conidia on diseased plant residues and in ginseng bed soil and seeds. Contaminated ginseng seeds and seedlings are the main sources of primary infection in new ginseng growing fields. In established ginseng growing fields, the primary infection sources are the pathogen-infected stems, leaves, and soil in ginseng sheds and beds. Overwintering conidia infect leaves and cause the disease to occur [5]. When conditions are favorable, the fungal pathogen spreads more extensively, further leading to large-scale disease occurrence. Thus, rapid, reliable early-stage pathogen detection is critical for effective disease management. Traditional diagnostics based on pathogen isolation, cultivation, and morphological identification are labor-intensive, time-consuming, and inherently insensitive. These methods are also prone to environmental interference, and many obligate parasites are difficult to culture in vitro [6].

Molecular detection technologies offer superior sensitivity, reproducibility, and speed compared with conventional approaches. Commonly used PCR-based methods include random amplified polymorphic DNA (RAPD), PCR-restriction fragment length polymorphism (PCR-RFLP), endpoint PCR, nested PCR, multiplex PCR, and quantitative real-time PCR (qPCR) [7,8,9]. Among these, qPCR is particularly advantageous for its exceptional sensitivity in quantifying trace pathogen DNA within complex biological matrices, such as host or environmental samples. Consequently, qPCR has become the preferred method for pathogen detection in clinical diagnostics, veterinary medicine, and agricultural science [10,11,12,13]. Numerous molecular assays have been developed for *Alternaria* spp. detection. Sharma and Tewari [14] employed RAPD-RFLP analysis to specifically detect *A. brassicae* among 29 strains infecting cruciferous crops. Achilonu et al. [15] developed a PCR-RFLP assay targeting the *Alt a1* gene region for rapid identification of pecan Alternaria black spot pathogens. Conventional PCR using ITS-specific primers has been used to identify *A. brassicae*, *A. brassicicola*, and *A. raphani* on crucifers [16], as well as *A. helianthi* on sunflowers [17,18]. Nested PCR, which is 10- to 100-fold more sensitive than conventional PCR, was applied for the rapid detection of *A. carthami* in safflower using universal primers ITS1/ITS4 for primary amplification and species-specific primers AcSPF/AcSPR for secondary amplification [19]. Gu et al. [20] reported a semi-nested PCR assay targeting a unique 251 bp fragment for specific diagnosis of potato early blight caused by *A. solani*. Multiplex PCR enables simultaneous detection of multiple targets in a single reaction; Kiran et al. [21] developed a multiplex assay for *A. brassicae*, *A. brassicicola*, and *Xanthomonas campestris* pv. *campestris* in crucifers, amplifying specific fragments of 586, 201, and 304 bp, respectively. Niu et al. [22] established a qPCR method for *A. solani* detection using cDNA as a template to target the stably expressed gene *jg1677*.

To date, little research has been reported on the molecular detection of the pathogens that cause Alternaria leaf and stem blight on ginseng. The single-tube nested PCR coupled with lateral-flow biosensor (STNPCR-LFBA) method, as described by Wei et al. [23], remains the only technique reported for the rapid identification of *A. panax*. This scarcity is largely attributable to the host specificity of *A. panax* to *P. ginseng* and its geographic restriction to East Asian cultivation regions, unlike globally distributed *Alternaria* pathogens of major staple crops that have prompted extensive methodological development. Therefore, the aim of this study was to establish a highly sensitive, specific, and accurate qPCR assay for *A. panax*. This qPCR assay further explored the relationship between the abundance of conidia or mycelia and the cycle threshold (Ct) value in soil. Finally, the feasibility of the qPCR assay was evaluated by analyzing artificially infected plant tissues and natural samples, including leaves, stems, seeds, and soil.

## 2. Materials and Methods

### 2.1. Strains and Cultivation Conditions

All fungal pathogens used in this study (Table S1) were isolated and preserved by the Plant Pathology Laboratory of Jilin Agricultural University. *Escherichia coli* DH5α (Tolobio, Shanghai, China) was used for plasmid construction. *Alternaria panax* was grown on V8 agar at 25 °C under a 12 h light/dark cycle for 14 d to induce conidiation. For DNA extraction, mycelia for DNA extraction were harvested from 7-day-old cultures grown on PDA at 25 °C. *E*. *coli* DH5α was propagated in LB medium or on LB plates for competent cell preparation.

### 2.2. Genomic DNA Extraction

Fifty milligrams of 7-day-old mycelia were gently harvested from PDA plates, and total DNA was recovered with the Fungal Genomic DNA Kit (Solarbio, Beijing, China). DNA from plant tissues and soil was isolated using commercial kits (TIANGEN, Beijing, China) according to the manufacturer’s protocols. DNA purity and concentration were assessed with a NanoDrop 2000 spectrophotometer (Thermo Fisher Scientific, Wilmington, NC, USA). Samples with A260/A280 ratios of 1.8–2.0 were retained for downstream PCR/qPCR, and the remainder were stored at −20 °C.

### 2.3. Specific Primer Design for the Conventional PCR and Quantitative Real-Time PCR (qPCR) Assays

A comparative genomics analysis based on protein sequences was performed to ensure species specificity and identify *Alternaria panax*-specific genes. Specifically, six species belonging to the genus *Alternaria* (*A. panax* GCA_019702505.1, *A. alternata* GCA_001642055.1, *A. tenuissima* GCA_004156035.1, *A. solani*, *A. brassicicola* and *A. longipes* from JGI database) and seven ginseng phytopathogens (*Phytophthora cactorum* GCA_016864655.1, *Rhizoctonia solani* GCA_016906535.1, *Sclerotinia sclerotiorum* GCF_000146945.2, *Fusarium oxysporum* GCF_013085055.1, *F. solani* GCA_023522795.1, *Botrytis cinerea* GCF_000143535.2, and *Ilyonectria robusta* GCF_021365365.1) with available gene annotations were selected for further investigation.

#### 2.3.1. Gene Family Analysis

A gene family is defined as a group of genes derived from a common ancestor through gene duplication or multiplication, whose members share similar structures and functions and encode similar protein products. Based on the protein sequences of the 13 samples, gene family analysis was performed using OrthoFinder (version 2.5.4). Potential homologous genes were identified using DIAMOND v2.1.16 with an E-value threshold of 0.001 (-e 0.001).

#### 2.3.2. Unique Gene Analysis

The unique genes and common genes from the 13 samples were separately merged into datasets. Redundans was applied to remove redundancy using the parameters -overlap 0.9 -identity 0.9. After deduplication, the common gene set and species-specific gene set for each species were obtained.

All the obtained species-specific genes were subjected to flanking sequence screening, and the purpose of this screening was to select suitable target genes for subsequent primer design, thereby providing a reliable basis for follow-up experimental operations relying on primer amplification. Primer specificity was validated in silico using Primer-BLAST (NCBI, www.ncbi.nlm.nih.gov/tools/primer-blast/, accessed on 27 June 2025) to exclude cross-reactivity with non-target *Alternaria* species, *Panax* host genome, and common soil fungi, combined with Primer Premier 6.0 for dimer/hairpin evaluation and thermodynamic optimization. The designed primers were then sent to Sangon Co., Ltd. (Shanghai, China) for synthesis.

### 2.4. Validation of Primer Specificity and Sensitivity for Conventional PCR

Primer specificity was first examined with standard endpoint PCR. Each 20 µL reaction comprised 10 µL Taq SuperMix (Novozyme, Nanjing, China), 1 µL each forward and reverse primer (10 µM), 1 µL template DNA and 7 µL nuclease-free water. Cycling began with 3 min at 95 °C, followed by 34 cycles of 95 °C for 10 s, primer-specific annealing temperature for 10 s and 72 °C for 30 s, and ended with a 5 min final extension at 72 °C. ddH_2_O was used as the negative control instead of template DNA. The PCR products were visualized on 1% (*w*/*v*) agarose gels. To determine the detection limit, *Alternaria panax* genomic DNA was 10-fold serially diluted (100–0.01 ng/µL). The lowest concentration yielding a visible band was defined as the detection limit. PCR products were separated on agarose gels and visualized using a non-toxic gel stain (Sangon Biotech, Shanghai, China). All tests were run in triplicate in three independent assays.

### 2.5. Standard Plasmid Preparation and Verification of Primers Specificity of qPCR

The specific PCR fragment of the unique gene from *Alternaria panax* was amplified with primers 1-2F/R and purified using a DNA gel extraction kit (Sangon, Shanghai, China). Amplicons were ligated into pUCm-T vectors (16 °C, 2 h) and introduced into *E. coli* DH5α. Positive colonies were selected on LB-ampicillin (100 μg/mL) and shaken in liquid LB at 37 °C, 200 rpm, overnight. Plasmids were extracted with a Mini Plasmid DNA Kit (Sangon, Shanghai, China) and Sanger-sequenced by the same supplier.

Primer specificity was tested against genomic DNA from *A. panax* and all other fungal isolates (Table S1). Quantitative PCR was subsequently run on a LightCycler 96 instrument (Roche Diagnostics, Mannheim, Germany). The qPCR reaction mixture contained 10.0 μL of 2 × Q5 SYBR qPCR Master Mix (Tolobio, Shanghai, China), 0.4 μL of each primer (10 μM), 2.0 μL of template DNA, and ddH_2_O to make up 20 μL. The qPCR reaction conditions were as follows: 95 °C for 180 s, followed by 40 cycles of 95 °C for 10 s and 60 °C for 30 s. After cycling, a melting curve was generated by heating to 95 °C, cooling to 65 °C for 15 s, and then heating to 97 °C at 5 °C/s, with fluorescence measured every 0.5 °C. Specificity of the qPCR primers was verified against genomic DNA from *A. panax* and all other fungal isolates tested. ddH_2_O served as the no-template negative control.

### 2.6. Sensitivity and Reproducibility of the qPCR Assays

A linear range of 1.65 × 10^2^–1.65 × 10^6^ copies/µL was prepared by 10-fold serial dilution of a quantified pUCm-T construct. Copy number was calculated from the plasmid mass concentration (ng/µL) and insert length (bp) using the following equation:

copies µL^−1^ = (6.02 × 10^23^ × ng/µL × 10^−9^)/(length × 660).

Each dilution was assayed in triplicate in three independent runs; Ct values were plotted against the log_10_ (copy number) to generate the calibration curve. To examine the reproducibility of qPCR, six replicates of every dilution within 1.65 × 10^1^–1.65 × 10^6^ copies/µL were tested on three separate days. Inter- and intra-run precision were expressed as the coefficient of variation (CV = SD/mean × 100%). The limit of detection (LOD) was determined by analyzing ten replicates of serially diluted plasmid DNA (down to single-copy level); the LOD was defined as the lowest concentration giving positive amplification in all ten reactions [24].

### 2.7. Establishing the Correlation Between the Abundance of Mycelia and Conidia of Alternaria panax and the Threshold Cycle (Ct) Value in Soil

Mycelial inoculum was prepared by inoculating five mycelial disks from 7-day-old PDA cultures into PDB and incubating at 25 °C, 170 rpm for 5 days. Mycelia were vacuum-filtered, lyophilized, and ground in liquid nitrogen. An amount of 1 g of mycelial powder was mixed with 9 g sterile soil (100 mg/g final concentration) and serially diluted 10-fold to obtain concentrations of 100, 10, 1, 1 × 10^−1^, 1 × 10^−2^, 1 × 10^−3^, 1 × 10^−4^, and 1 × 10^−5^ mg/g. For conidia inoculation, the conidia suspension of strain SD3-3 (10^3^ conidia/mL) was added to soil to achieve densities of 0, 50, 100, 200, and 500 conidia/g. After incubation at 25 °C for 12 h, 0.25 g of soil was collected and deposited at −80 °C for future use. Soil DNA was extracted using a soil DNA extraction kit (TIANGEN, Beijing, China) and analyzed by qPCR in triplicate across three independent experiments.

### 2.8. Quantification of Alternaria panax in Artificially Inoculated Leaf, Stem and Seed Samples

Leaves were excised from 3-year-old ginseng, surface-sterilized (75% ethanol, 2 min; 2% NaOCl, 1 min; three sterile-water rinses), and air-dried. Conidial suspensions were prepared from strain SD3-3 cultured on V8 medium at 25 °C for 14 days under a 12 h light/12 h dark photoperiod. Conidia were harvested using a sterile spreader, washed twice with sterile distilled water, resuspended in 5 mL of sterile distilled water, filtered through two layers of Miracloth, and enumerated using a hemocytometer. Twenty microliters of conidia suspension (1 × 10^5^ conidia/mL) was inoculated onto individual healthy ginseng leaves. The inoculated ginseng leaves were kept in a sterile, moist chamber and incubated at 25 °C for different cultivation periods. For stem inoculation, 8 cm long detached stems from three-year-old ginseng plants were surface-disinfected and inoculated using the methods described above. DNA was extracted from inoculated leaves and stems at designated time points post-inoculation and quantified by qPCR.

For seed inoculation, freshly harvested ginseng seeds were surface-sterilized and immersed in conidial suspension (1 × 10^5^ conidia/mL) at 25 °C. Samples (5 seeds per replicate, 3 replicates per time point) were collected at 0 h, 3 h, 6 h, 12 h, 24 h, 48 h, and 72 h. Seeds were dissected into seed coats and kernels, rinsed, and snap-frozen in liquid nitrogen. DNA from each seed part (0.1 g) was extracted using the CTAB method and analyzed by qPCR in triplicate across three independent experiments.

### 2.9. Detection and Quantification of Alternaria panax in Naturally Grown Leaf, Stem, Soil, and Seed Samples from Ginseng-Growing Areas

A total of 286 samples, including leaves, stems, seeds and soil samples, were collected from different ginseng-growing areas, primarily in Jilin Province (Table S2). Disease severity of leaf and stem samples was rated as described previously [25]. The disease grading criteria was assessed using a 0–9 scale based on the percentage of leaf/stem area affected: 0 = no symptoms; 1 = <5% leaf/stem area affected; 3 = 6–10% leaf/stem area affected; 5 = >11–20% leaf/stem area affected; 7 = >21–50% leaf/stem area affected; and 9 >50% leaf/stem area affected. Plant tissues and seeds were washed with tap water several times before DNA isolation. Seeds were dissected into coat and kernel, then pulverized in liquid nitrogen. DNA of all the samples was extracted using plant and soil genomic DNA extraction kits, following the respective instructions. Quantitative PCR was performed on a LightCycler 96 as described above, with template-free negative controls included in each run. Pathogen DNA concentration was calculated from Ct values using the standard curve generated in Section 2.6. All samples were analyzed in triplicate.

## 3. Results

### 3.1. Design and Development of an Alternaria panax-Specific qPCR Assay

**Figure 1:**
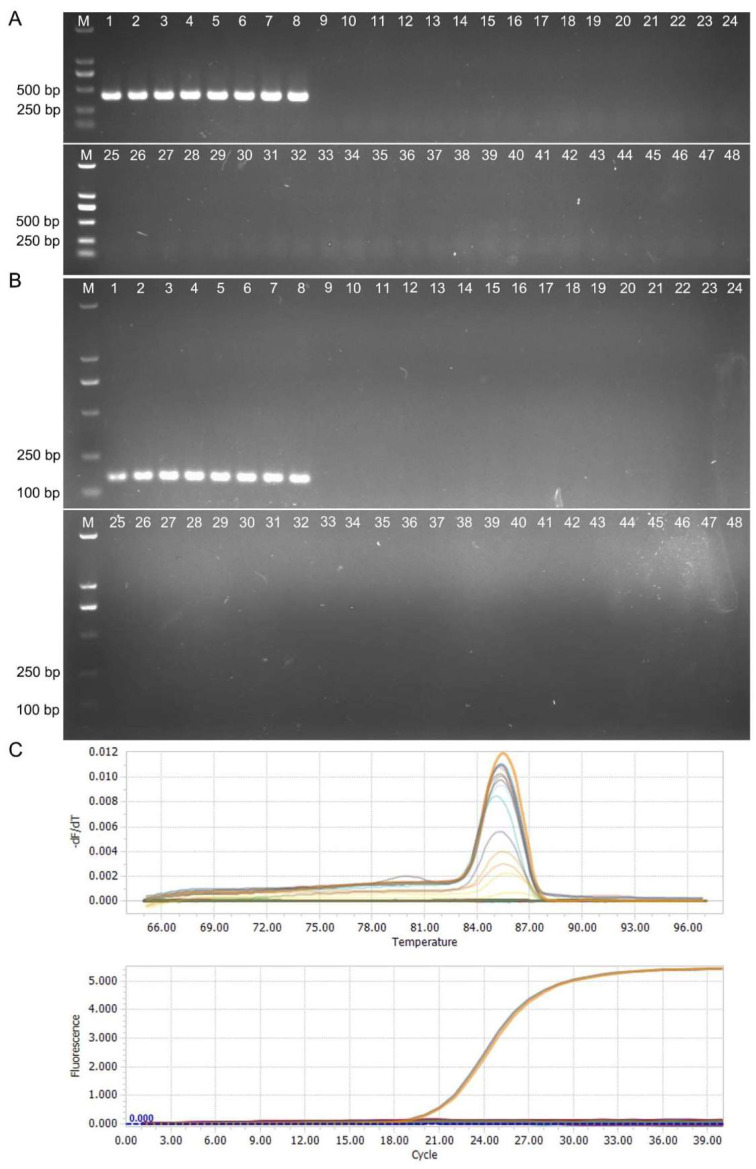
Specificity of primers targeting *Alternaria panax*. (**A**) Conventional PCR (primer set 1-2F/R) tested against 47 isolates. Lanes 1–8: *A. panax* from ginseng (1–5), eleuthero (6), ginseng (7) and sanqi (8); Lanes 9–14: *A. tenuissima* from ginseng; Lanes 15–20: *A. alternata* from ginseng; Lane 21: *A. solani* (eggplant); Lane 22: *A. brassicicola* (Chinese cabbage); Lanes 23–25: *A. alternata* (radish), *A. tenuissima* (radish), *A. alternata* (Chinese cabbage); Lane 26: *A. jacinthicola* (marigold); Lane 27: *A. helianthinficiens* (cosmos); Lane 28: *A. yaliinficiens* (tobacco); Lane 29: *A. longipes* (tobacco); Lane 30: *A. alternata* (eleuthero); Lane 31: *A. tenuissima* (tobacco); Lane 32: *A. alternata* (tobacco); Lanes 33–39: *Botrytis cinerea* (ginseng); Lane 40: *B. fabae* (ginseng); Lanes 41–47: *Phytophthora cactorum*, *Colletotrichum panacicola*, *Ilyonectria robusta*, *Rhizoctonia solani*, *Fusarium oxysporum*, *P. cactorum* and *Sclerotinia sclerotiorum* (all from ginseng); Lane 48: ddH_2_O. (**B**) qPCR specificity (primer set Q2-1F/R); Lane order as in (**A**). (**C**) Melting curve (upper) and amplification curve (lower) of primer set Q2-1F/R. Melting curve analysis showed that only *A. panax* samples produced single-peak melting curves. Different colors represent distinct detection channels.

Unique genes of *A. panax* were identified via comparative-genome analysis among ginseng pathogenic fungi. One unique gene (GenBank ID: KAG9184916.1) was selected for specific primer design (Figure S1). Conventional PCR using primers 1-2F/R (1-2F: 5′-ATACGGGTGCTGGGTTGA-3′, 1-2R: 5′-ACCTCGCAATCACGGTAC-3′) was performed on *A. panax* isolates from different locations, yielding specific amplicons for *A. panax* but no amplification for other phytopathogenic fungi (Figure 1A). Additionally, qPCR primers Q2-1F/R (Q2-1-F: 5′-ATCGCTGTGGATGCTTGT-3′, Q2-1R: 5′-CGTCCGACCAATATGGGTATC-3′), designed based on the specific fragment amplified by primers 1-2F/R, also demonstrated specificity in conventional PCR (Figure 1B). Subsequently, all tested strains were subjected to qPCR analysis using primers Q2-1F/R. As shown in Figure 1C, these primers generated a single specific melting curve peak for *A. panax* DNA templates, with no corresponding peaks for other pathogenic fungi. Consistently, only *A. panax* strains generated amplification curves (Figure 1C).

### 3.2. Sensitivity, Reproducibility and Standard Curve Establishment of the Alternaria panax qPCR Assay

**Figure 2:**
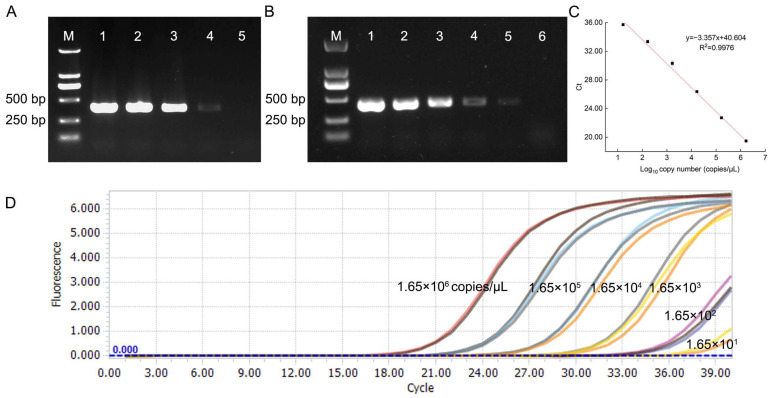
Sensitivity of conventional PCR and qPCR. (**A**) Conventional PCR sensitivity. The genomic DNA of *Alternaria panax* was diluted to five concentrations and used as template: Lane 1: 100 ng/μL; Lane 2: 10 ng/μL; Lane 3: 1 ng/μL; Lane 4: 0.1 ng/μL; Lane 5: 0.01 ng/μL. (**B**) The plasmid DNA was diluted to six concentrations and used as template: Lane 1: 100 ng/μL; Lane 2: 10 ng/μL; Lane 3: 1 ng/μL; Lane 4: 0.1 ng/μL; Lane 5: 0.05 ng/μL; Lane 6: 0.01 ng/μL. (**C**) Standard curve of qPCR assays. The standard plasmid was serially 10-fold diluted from 1.65 × 10^−2^ to 1.65 × 10^6^ copies/μL and used as a template. X-axis: The logarithm of plasmid DNA concentration; Y-axis: Ct values. (**D**) Representative qPCR amplification curves obtained with the same plasmid dilution series (1.65 × 10^−2^–1.65 × 10^6^ copies/μL).

*A. panax* genomic DNA was ten-fold serially diluted (100–0.01 ng/μL) and subjected to conventional PCR with primers 1-2F/1-2R. The results showed that the limit of detection (LOD) for conventional PCR was 0.1 ng/μL using genomic DNA as template, and 0.05 ng/μL using plasmid DNA (Figure 2A,B). A qPCR standard curve was generated using the standard plasmid ranging from 1.65 × 10^1^ to 1.65 × 10^6^ copies/μL as the template. The standard curve was established by plotting the logarithm of plasmid DNA copy number against the corresponding Ct values. The standard curve equation, y = −3.357x + 40.604, exhibited a strong linear relationship (R^2^ = 0.9976) (Figure 2B). The amplification efficiency was calculated based on the standard curve (slope = −3.357) and was determined to be 98.6%, which falls within the acceptable range of 90–110%. The LOD experiment showed that the lowest concentration yielding a positive signal in ≥95% of replicates was 1.65 × 10^1^ copies/μL, whereas 20% of reactions failed at 1.65 × 10^0^ copies/μL (Figure 2C, Table S3). Therefore, according to the LOD establishment criteria, the limit of qPCR detection was, therefore, determined to be 1.65 × 10^1^ copies/μL.

Reproducibility was evaluated by analyzing six replicates of each point in the 1.65 × 10^1^–1.65 × 10^6^ copies/μL plasmid series under identical qPCR conditions. The coefficients of variation with concentrations ranging from 1.65 × 10^1^ to 1.65 × 10^6^ copies/μL were 0.33%, 0.04%, 0.56%, 1.06%, 0.606%, and 0.52%, respectively. Since all these values were below 2% (from technical replicates), the qPCR assay showed excellent reproducibility (Table 1).

### 3.3. Correlation Between Alternaria panax Inoculum Concentration in Soil and Ct Values

**Figure 3:**
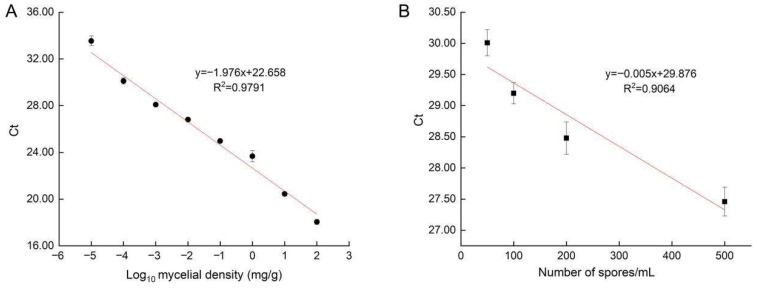
Relationship between soil inoculum density and qPCR Ct value. (**A**) Correlation equation between mycelium content and Ct value in soil. X-axis: the logarithm of mycelium content; Y-axis: Ct values. (**B**) Correlation equation between conidia concentration and Ct value in soil. X-axis: conidia concentration; Y-axis: Ct values.

Sterile soil samples were supplemented with *A. panax* mycelia at concentrations of 100, 10, 1, 10^−1^, 10^−2^, 10^−3^, 10^−4^, and 10^−5^ mg/g for DNA extraction. Sterile soil without mycelia and ddH_2_O served as negative controls. qPCR assays showed that the mycelia of *A. panax* were undetectable at 1 × 10^−5^ mg/g. Ct values (*y*-axis) exhibited a strong linear correlation with the logarithm of mycelial density (*x*-axis) (Figure 3A), with the equation y = −1.976x + 22.658 (R^2^ = 0.9791). In conidia-inoculated soil experiments, *A. panax* was detected in all the inoculation concentrations of 50, 100, 200 and 500 conidia/g soil. A linear relationship was established between Ct values (y-axis) and the *A. panax* conidia numbers (x-axis), with the equation y = −0.005x + 29.876 and R^2^ = 0.9064 (Figure 3B).

### 3.4. Quantification of Alternaria panax in Artificially Inoculated Leaves, Stems and Seeds

**Figure 4:**
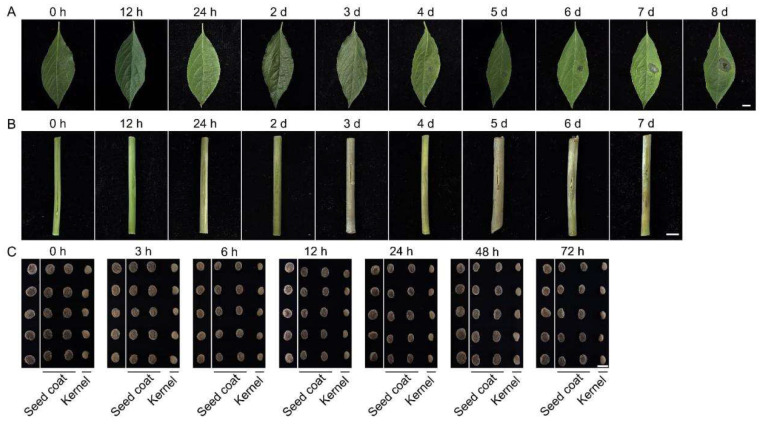
Symptom development after artificial inoculation of ginseng leaves (**A**), stems (**B**) and seeds (**C**) with *Alternaria panax*. The 10 seed coats and 5 kernels were derived from the same 5 seeds, with each seed dissected into two seed coat halves and one kernel (**C**). Bars = 1 cm.

The sensitivity of the developed qPCR assay was validated using artificially infected ginseng leaves, stems and seeds. For detached ginseng leaf samples, at 12 h post-inoculation, the Ct value was 27.80 ± 0.11 and the calculated DNA concentration was 1.10 ± 0.08 fg/μL (Table 2). No symptoms were observed up to 3 days post-inoculation, but Ct values progressively decreased while DNA concentrations increased (Figure 4A). Symptoms became apparent at 4 days post-inoculation, with *A. panax* DNA concentration reaching 37.49 ± 5.17 fg/μL. By day 8, lesions had enlarged and darkened, with DNA concentration reaching 572.26 ± 26.26 fg/μL. Similar detection profiles were observed for stem inoculation. Although visible symptoms were absent until day 4 post-inoculation (Figure 4B), Ct values progressively decreased (Table 2). By day 7, the Ct value reached 21.80 ± 0.11, corresponding to a DNA concentration of 67.19 ± 5.22 fg/μL. In artificial seed-inoculation assays, no visible symptoms were observed on seed surfaces throughout the 72 h post-inoculation period (Figure 4C). However, Ct values progressively declined with extended incubation. Notably, Ct values for seed coats were consistently lower than those for kernels (Table 2).

### 3.5. Detection and Quantification of Alternaria panax DNA in Naturally Grown Plant Tissues and Soil Samples from Ginseng-Growing Areas

Plant tissues (leaves and stems), seeds and soil were primarily sampled from ginseng-growing areas of Jilin Province. After DNA isolation, all these samples were amplified by qPCR using specific primers Q2-1F/R. ddH_2_O served as the negative control in each run. qPCR analysis of leaves and stems revealed that Ct values generally correlated with visually assigned disease severity grades. However, some discrepancies were observed in several samples, where low Ct values (indicating high *A. panax* biomass) were detected in visually less-severe disease grades. For instance, the grade-3 sample FS34-L exhibited a Ct value of 33.78 ± 1.05, whereas grade-1 samples FS38-L and FS48-L showed Ct values of 33.58 ± 0.93 and 33.67 ± 0.45, respectively. Furthermore, detectable *A. panax* levels were observed in numerous grade-0 samples, which were visually healthy and lesion-free according to disease grading criteria (Table 3). Specifically, *A. panax* was detected at 12.12% of grade-0 leaf tissues and 14.29% of grade-0 stem tissues. These results demonstrate that this qPCR assay provides a more objective and sensitive assessment of pathogen load compared with visual disease grading.

Across all seed samples, the detection rate of *A. panax* was 23.73% (Table 4). Ct values for seed coats were consistently lower than those for kernels, indicating higher pathogen loads in seed coats. In several samples, *A. panax* was detected exclusively in seed coats while corresponding kernels tested negative, indicating that the fungus had not yet penetrated the kernel (Table 4). Additionally, *A. panax* was detected in 14.46% of soil samples collected from different ginseng-growing areas (Table 5).

## 4. Discussion

At present, PCR-based molecular detection methods have largely replaced conventional culture-dependent techniques for identification of fungal pathogens, with quantitative PCR (qPCR) emerging as a particularly prominent and trustworthy diagnostic approach. In this study, we developed a qPCR assay for *Alternaria panax* with high specificity, sensitivity, and reproducibility. The feasibility of the established in various samples, including plant tissues, seeds and soil.

Traditional diagnostic methods have historically been used for ginseng disease diagnosis [26,27,28,29,30]. However, their labor-intensive and time-consuming nature often requires weeks to months to obtain results, restricting rapid and accurate disease detection. Over the past five years, molecular methods have been increasingly explored for this purpose. PCR-based techniques, including conventional PCR, multiplex PCR, and qPCR, have been reported for soil-borne pathogens of ginseng such as *Phytophthora cactorum*, *Fusarium oxysporum*, *Ilyonectria* spp., *Cylindrocarpon destructans*, *Rhizoctonia solani*, and *Sclerotinia ginseng* [31,32,33,34,35]. Additionally, an improved PCR-Nucleic Acid Sensor (PCR-NAS) technique has been established for *Botrytis cinerea*, a common foliar pathogen of ginseng [36]. To date, only one study has reported a single-tube nested PCR-lateral flow biosensor assay (STNPCR-LFBA) for the detection of *A. panax* [23]. Under their respective optimized conditions, the qPCR assay achieved an analytical detection limit of 0.074 fg/µL, compared to 0.01 pg/μL reported for STNPCR-LFBA. This represents an apparent 180-fold improvement in analytical sensitivity, though direct comparison is moderated by differences in detection principles and sensitivity definitions.

Detecting infections in plants at an early stage is advantageous for efficient disease control. The application of various molecular detection techniques has greatly improved the identification of distinct fungal plant pathogens [37]. In this study, the results of artificial leaf and stem inoculation showed that, even during the asymptomatic stage, the Ct values of the qPCR detection results gradually decreased with increasing post-inoculation time (Table 2). This indicated rising *A. panax* DNA levels, implying active fungal pathogen proliferation and spread during the asymptomatic stage. During seed assays, *A. panax* was detected in both the seed coat and the kernel. Ct values decreased with the extension of post-inoculation time, with Ct values for the seed coat remaining lower than those for the kernel. These results demonstrate that qPCR is substantially more sensitive than conventional methods, providing a foundation for early disease forecasting and management.

Soil represents a challenging substrate for accurate microbial quantification due to heterogeneous pathogen distribution, variable extraction efficiency across soil chemistries, and the presence of PCR inhibitors such as humic acids and plant secondary metabolites. DNA extraction efficiency constitutes a critical determinant of qPCR accuracy: incomplete pathogen cell lysis, DNA adsorption to soil colloids, or co-precipitation with organic matter can reduce template yield, while soil-derived inhibitors may suppress Taq polymerase activity—both resulting in elevated Ct values that underestimate actual pathogen loads. The present qPCR assay was validated using purified *A. panax* DNA standards, without incorporation of internal controls for extraction efficiency or amplification inhibition. This constitutes a recognized limitation for environmental sample analysis. To partially mitigate this, we established a dose-dependent relationship between spiked conidia concentrations and Ct values in artificially inoculated soil samples, validating quantitative reliability under controlled matrix conditions. Nevertheless, future optimization of this assay will require: (i) incorporation of internal controls to monitor extraction and amplification efficiency; and (ii) establishment of robust correlations between molecular quantification and field disease incidence under local agroecological conditions. The present study provides the methodological foundation for *A. panax* detection; however, site-specific calibration is essential for reliable field deployment.

The limit of detection was established at ~16.5 copies/μL (Ct value ~35) according to standard LOD criteria, using purified *A. panax* DNA standards. However, an important consideration warrants discussion regarding assay application in complex samples. The complexity of samples, particularly soil samples, may lead to DNA extracts containing PCR inhibitors (humic acids, polyphenols), ultimately reducing amplification efficiency and increasing Ct values. This necessitates careful interpretation of results from environmental samples compared to pure culture standards. For large-scale applications, the correlation between determined Ct values and actual disease occurrence/severity requires further investigation across complete growing seasons. This is a long-term validation task. In this study, we have constructed and validated the qPCR assay; ongoing work focuses on establishing the epidemiological correlation between molecular detection and field disease symptoms. Nevertheless, the present assay supports two key applications: (i) pre-planting soil assessment to identify pathogen-free sites or guide rotation decisions; and (ii) in-season monitoring to track inoculum dynamics and inform management timing.

Application of the qPCR assay to natural samples revealed frequent detection of *A. panax* in visually healthy, symptomless leaf and stem tissues (grade 0), highlighting superior sensitivity over culture-based techniques. It is important to recognize that qPCR detects pathogen DNA irrespective of its physiological state; consequently, positive amplification in asymptomatic tissues may reflect early-stage infection, latent colonization, or residual DNA from non-viable propagules. Among seeds from different locations in Jilin Province, 23.73% harbored *A. panax*, while 14.46% of soil samples from ginseng-growing areas contained the pathogen. Notably, several grade-1 and grade-3 samples showed marked discrepancies between visual disease severity and corresponding Ct values, with low-grade tissues often yielding unexpectedly low Ct values and occasional high-grade samples showing elevated Ct values. Spatial heterogeneity of pathogen distribution within tissues further contributes to this variability. These observations collectively indicate that qPCR quantifies pathogen DNA burden rather than active disease intensity and should be interpreted in conjunction with phenotypic assessment for comprehensive disease diagnosis. Additionally, visual disease assessment, while practical for field applications, may suffer from subjectivity due to variations in observer experience, lighting conditions, or lesion type. In contrast, qPCR-based Ct values provide objective, reproducible quantification of *A. panax* DNA, independent of symptom appearance or tissue type. Due to the narrow geographical scope of sampling (with the majority of isolates originating from Jilin Province, China), the feasibility of this qPCR assay may be compromised when applied to other ginseng cultivation areas with divergent environmental conditions (e.g., soil physicochemical properties, climate, microbial communities). The optimization of this method would require site-specific validation to account for regional variations in sample matrix effects.

Reducing primary infection sources is a vital measure for integrated control of ginseng Alternaria leaf and stem blight. In established ginseng fields, the primary sources of *A. panax* are diseased plant residues and soil-borne pathogens in the soil, while in new fields, the primary sources are pathogen-carrying seeds and seedlings. Early diagnosis of pathogen contamination in soil and seeds is therefore crucial. However, seeds are often asymptomatic, and pathogens in the soil require isolation and cultivation for identification [38]. The qPCR method developed here offers a rapid, sensitive, and reliable approach for composite testing of bulk seed lots, enabling accurate estimation of low-level pathogen prevalence from a single reaction and minimizing risks associated with sowing infected seed. This highly specific diagnostic tool contributes to the precise determination of threshold inoculum concentrations required for seed-to-seedling transmission and can be extended to trace pathogen flux on contaminated equipment or processing surfaces [39]. Utilizing this molecular detection method for pre-planting seed and soil analysis facilitates the selection of pathogen-free planting sites and materials, thereby averting economic losses from disease.

## 5. Conclusions

A quantitative real-time PCR assay specific to *Alternaria panax* has been established in this study. The reliability of the established assay was verified by detecting *A*. *panax* in artificially inoculated leaves, stems, and seeds. Additionally, a correlation was constructed between the abundance of *A. panax* mycelia and conidia and the Ct value in soil. The established method was then used to detect *A*. *panax* on naturally infected plants and field soils. This rapid, sensitive, and specific assay provides a robust tool for the early screening of planting material such as seeds and seedlings, early disease diagnosis and certification of pathogen-free soils for ginseng cultivation. Furthermore, this method lays the groundwork for investigating the infection cycle and epidemic dynamics of Alternaria leaf and stem blight, thereby supporting the sustainable management of this disease in ginseng.

## Figures and Tables

**Table 1:** Assessment of qPCR reproducibility.

Plasmid Concentration (Copies/μL)	Intra-Assay Replicate			Inter-Assay Replicate		
	Mean Ct	SD	CV (%)	Mean Ct	SD	CV (%)
1.65 × 10^6^	19.48	0.063	0.33	19.55	0.320	1.67
1.65 × 10^5^	22.71	0.103	0.04	23.01	0.184	0.79
1.65 × 10^4^	26.37	0.151	0.56	26.43	0.175	0.66
1.65 × 10^3^	30.31	0.324	1.06	30.32	0.215	0.71
1.65 × 10^2^	33.35	0.204	0.61	33.28	0.291	0.87
1.65 × 10^1^	35.71	0.188	0.52	36.11	0.231	0.63

Note: SD, standard deviation; CV, coefficient of variation.

**Table 2:** Detection and quantification of *Alternaria panax* in artificially inoculated samples by qPCR assays.

Leaf			Stem			Seed				
Inoculation Time	Ct Value	DNA Concentration (fg/μL)	Inoculation Time	Ct Value	DNA Concentration (fg/μL)	Inoculation Time	Ct Value		DNA Concentration (fg/μL)	
							Seed Coat	Kernel	Seed Coat	Kernel
0 h	30.53 ± 0.50	0.17 ± 0.05	0 h	30.24 ± 0.10	0.21 ± 0.01	0 h	33.96 ± 0.29	37.68 ± 0.23	0.02 ± 0.003	0.001 ± 0.00
3 h	30.28 ± 0.69	0.21 ± 0.09	3 h	30.16 ± 0.02	0.22 ± 0.003	3 h	29.62 ± 0.21	34.77 ± 0.14	0.32 ± 0.05	0.009 ± 0.00
6 h	29.80 ± 0.41	0.28 ± 0.07	6 h	29.87 ± 0.36	0.27 ± 0.06	6 h	28.41 ± 0.30	32.36 ± 0.51	0.73 ± 0.147	0.05 ± 0.02
9 h	29.18 ± 0.63	0.45 ± 0.18	24 h	28.39 ± 0.25	0.74 ± 0.13	12 h	26.02 ± 0.29	30.51 ± 0.26	3.76 ± 0.743	0.17 ± 0.03
12 h	28.08 ± 0.21	0.91 ± 0.14	48 h	27.77 ± 0.34	1.14 ± 0.26	24 h	25.67 ± 0.37	29.17 ± 0.57	4.86 ± 1.248	0.46± 0.18
24 h	27.80 ± 0.11	1.10 ± 0.08	3 d	26.74 ± 0.33	2.34 ± 0.53	48 h	24.31 ± 0.44	27.75 ± 0.51	12.44 ± 3.72	1.19 ± 0.38
48 h	27.27 ± 0.11	1.58 ± 0.11	4 d	25.68 ± 0.18	4.71 ± 0.57	72 h	24.05 ± 0.35	26.57 ± 0.42	14.64 ± 3.55	2.64 ± 0.76
3 d	26.80 ± 0.13	2.18 ± 0.19	5 d	23.63 ± 0.23	19.22 ± 2.85	ddH_2_O	—	—	—	—
4 d	22.66 ± 0.20	37.49 ± 5.17	6 d	22.66 ± 0.20	37.39 ± 5.17					
5 d	22.06 ± 0.15	56.31 ± 5.85	7 d	21.80 ± 0.11	67.19 ± 5.22					
6 d	21.90 ± 0.17	63.30 ± 7.65	ddH_2_O	—	—					
7 d	21.63 ± 0.14	75.57 ± 7.23								
8 d	18.69 ± 0.08	572.26 ± 26.26								
ddH_2_O	—	—								

Note: DNA concentration was calculated based on target amplicon.

**Table 3:** Detection and quantification of *Alternaria panax* in natural ginseng leaves and stems by qPCR assays.

Sample ID-Leaf	Disease Grading	Ct Value	Sample ID-Stem	Disease Grading	Ct Value
JY1-L	1	34.02 ± 0.46	JY1-ST	1	34.33 ± 0.26
JY2-L	0	—	TH1-ST	0	—
JY3-L	0	—	TH2-ST	0	—
JY4-L	0	37.43 ± 0.12	FS1-ST	3	31.23 ± 0.68
JY5-L	0	35.36 ± 0.38	FS2-ST	5	29.28 ± 0.56
JY6-L	1	33.55 ± 1.22	FS3-ST	3	31.86 ± 0.19
JY7-L	0	—	FS4-ST	0	—
TH1-L	7	25.16 ± 0.05	FS5-ST	0	—
TH2-L	7	25.83 ± 0.81	FS6-ST	0	35.45 ± 0.11
TH3-L	3	31.36 ± 0.14	FS7-ST	0	—
TH4-L	5	27.66 ± 0.01	FS8-ST	1	33.89 ± 0.51
TH5-L	3	30.87 ± 0.46	FS9-ST	3	30.14 ± 0.22
FS1-L	0	—	FS10-ST	0	36.58 ± 0.47
FS2-L	3	31.38 ± 0.27	FS11-ST	1	34.48 ± 0.74
FS3-L	0	—	FS12-ST	0	—
FS4-L	0	—	FS13-ST	0	—
FS5-L	3	30.59 ± 0.16	FS14-ST	9	25.78 ± 0.11
FS6-L	0	—	FS15-ST	1	34.36 ± 0.22
FS7-L	0	—	FS16-ST	7	27.61 ± 0.43
FS8-L	0	—	FS17-ST	7	27.44 ± 0.78
FS9-L	0	37.46 ± 0.35	FS18-ST	3	32.56 ± 0.11
FS10-L	0	35.24 ± 0.85	FS19-ST	0	—
FS11-L	0	35.96 ± 0.81	FS20-ST	5	28.56 ± 0.29
FS12-L	0	35.85 ± 0.46	FS21-ST	5	28.99 ± 0.18
FS13-L	0	36.14 ± 0.63	FS22-ST	3	30.24 ± 0.81
FS14-L	0	—	FS23-ST	0	—
FS15-L	0	36.86 ± 0.46	FS24-ST	7	27.38 ± 0.34
FS16-L	1	34.71 ± 0.67	FS25-ST	3	32.03 ± 0.39
FS17-L	1	34.20 ± 0.39	FS26-ST	3	32.48 ± 0.42
FS18-L	3	30.81 ± 0.22	FS27-ST	0	—
FS19-L	0	—	FS28-ST	0	—
FS20-L	5	28.38 ± 0.76	FS29-ST	7	27.32 ± 0.47
FS21-L	3	31.51 ± 1.01	FS30-ST	3	31.43 ± 0.19
FS22-L	5	29.41 ± 0.22	FS31-ST	0	—
FS23-L	5	28.71 ± 0.34	FS32-ST	5	29.31 ± 0.29
FS24-L	1	34.70 ± 0.27	FS33-ST	0	—
FS25-L	1	34.82 ± 0.29	FS34-ST	9	24.19 ± 1.01
FS26-L	0	—	FS35-ST	1	34.67 ± 0.31
FS27-L	9	21.50 ± 0.39	FS36-ST	1	33.71 ± 0.41
FS28-L	5	27.61 ± 0.46	FS37-ST	5	29.47 ± 0.47
FS29-L	5	29.68 ± 1.42	FS38-ST	0	—
FS30-L	3	30.89 ± 0.67	NG1-ST	5	28.17 ± 0.28
FS31-L	0	—	NG2-ST	3	31.47 ± 0.02
FS32-L	0	37.23 ± 0.62	NG3-ST	7	26.76 ± 0.85
FS33-L	1	34.77 ± 0.32	NG4-ST	5	28.44 ± 1.02
FS34-L	3	33.78 ± 1.05	NG5-ST	5	29.03 ± 0.73
FS35-L	0	36.55 ± 0.31	NG6-ST	0	34.56 ± 0.59
FS36-L	1	33.22 ± 0.65	NG7-ST	0	—
FS37-L	1	32.75 ± 0.44	NG8-ST	0	—
FS38-L	1	33.58 ± 0.93	NG9-ST	7	27.42 ± 0.48
FS39-L	0	—	NG10-ST	5	29.17 ± 0.06
FS40-L	0	35.17 ± 0.47	NG11-ST	3	31.48 ± 0.35
FS41-L	0	—	NG12-ST	3	32.45 ± 0.62
FS42-L	1	34.92 ± 0.97	NG13-ST	1	34.01 ± 0.83
FS43-L	7	27.12 ± 0.48	NG14-ST	0	—
FS44-L	5	28.89 ± 0.42	NG15-ST	5	29.19 ± 0.24
FS45-L	5	27.58 ± 0.94	NG16-ST	0	35.24 ± 0.81
FS46-L	5	28.43 ± 0.86	ddH_2_O	—	—
FS47-L	0	36.09 ± 0.22			
FS48-L	1	33.67 ± 0.45			
FS49-L	0	36.54 ± 0.34			
FS50-L	5	29.37 ± 0.65			
FS51-L	5	30.12 ± 0.39			
FS52-L	3	31.44 ± 0.38			
FS53-L	0	—			
FS54-L	9	23.82 ± 0.75			
FS55-L	0	—			
FS56-L	3	32.87 ± 0.67			
NG1-L	3	30.97 ± 0.28			
NG2-L	1	32.77 ± 1.04			
NG3-L	1	34.11 ± 0.42			
NG4-L	1	34.07 ± 0.73			
NG5-L	7	23.95 ± 0.46			
NG6-L	0	35.33 ± 0.05			
NG7-L	7	27.15 ± 0.77			
NG8-L	0	38.19 ± 0.62			
NG9-L	1	33.33 ± 0.68			
NG10-L	3	30.79 ± 0.05			
NG11-L	5	27.66 ± 0.86			
NG12-L	3	31.53 ± 0.42			
NG13-L	3	32.04 ± 0.73			
NG14-L	0	38.25 ± 0.42			
NG15-L	9	25.51 ± 0.27			
NG16-L	5	28.75 ± 0.45			
NG17-L	1	33.33 ± 0.29			
NG18-L	1	34.15 ± 0.25			
NG19-L	3	31.55 ± 0.49			
ddH_2_O	—	—			

Note: according to LOD validation, a Ct value < 35.45 was considered indicative of *A. panax* detection.

**Table 4:** Detection and quantification of *Alternaria panax* in natural ginseng seed samples by qPCR assays.

Sample ID	Ct Value		Sample ID	Ct Value		Sample ID	Ct Value	
	Seed Coat	Kernel		Seed Coat	Kernel		Seed Coat	Kernel
FS1-SE	—	—	FS21-SE	32.19 ± 0.15	36.42 ± 0.33	WQ2-SE	—	—
FS2-SE	—	—	FS22-SE	—	—	WQ3-SE	—	—
FS3-SE	30.89 ± 0.34	34.99 ± 1.36	FS23-SE	—	—	WQ4-SE	38.17 ± 0.62	—
FS4-SE	29.87 ± 0.31	—	FS24-SE	—	—	WQ5-SE	37.62 ± 0.27	39.44 ± 0.35
FS5-SE	—	—	FS25-SE	—	—	AT1-SE	37.32 ± 0.63	37.99 ± 0.49
FS6-SE	—	—	FS26-SE	—	—	DH1-SE	31.76 ± 0.08	36.74 ± 0.37
FS7-SE	—	—	FS27-SE	—	—	JA1-SE	29.38 ± 0.26	—
FS8-SE	—	—	FS28-SE	—	—	JA2-SE	29.58 ± 0.22	—
FS9-SE	31.59 ± 0.36	33.97 ± 0.24	FS29-SE	—	—	JA3-SE	—	—
FS10-SE	29.95 ± 0.37	—	FS30-SE	36.63 ± 0.02	—	JA4-SE	36.53 ± 0.29	—
FS11-SE	—	—	FS31-SE	—	—	JA5-SE	36.49 ± 0.66	38.39 ± 0.72
FS12-SE	—	—	FS32-SE	35.82 ± 0.16	38.80 ± 0.42	DQ1-SE	37.75 ± 0.31	—
FS13-SE	35.35 ± 0.05	—	FS33-SE	38.46 ± 0.37	39.54 ± 0.22	HD1-SE	—	—
FS14-SE	—	—	FS34-SE	34.94 ± 0.24	36.39 ± 0.30	KD1-SE	38.13 ± 0.81	—
FS15-SE	37.97 ± 0.11	—	FS35-SE	37.31 ± 0.44	—	KD2-SE	34.13 ± 0.81	—
FS16-SE	32.08 ± 0.26	—	FS36-SE	—	—	KD3-SE	29.81 ± 0.28	—
FS17-SE	—	—	FS37-SE	—	—	KD4-SE	—	—
FS18-SE	—	—	FS38-SE	38.25 ± 0.02	—	KD5-SE	—	—
FS19-SE	38.49 ± 0.07	—	FS39-SE	36.25 ± 0.19	—	QY1-SE	—	—
FS20-SE	34.37 ± 0.18	—	WQ1-SE	—	—	ddH_2_O	—	—

Note: according to LOD validation, a Ct value < 35.45 was considered indicative of *A. panax* detection.

**Table 5:** Detection and quantification of *Alternaria panax* in natural soil samples by qPCR assays.

Sample ID	Ct Value	Sample ID	Ct Value	Sample ID	Ct Value	Sample ID	Ct Value
HOQ1-SO	—	WQ2-SO	36.94 ± 0.44	FS12-SO	—	FS34-SO	—
HOQ2-SO	—	WQ3-SO	—	FS13-SO	35.99 ± 0.45	FS35-SO	30.82 ± 0.42
HOQ3-SO	—	WQ4-SO	—	FS14-SO	—	FS36-SO	36.16 ± 0.78
HOQ4-SO	—	WQ5-SO	—	FS15-SO	30.71 ± 0.38	FS37-SO	—
DD1-SO	—	WQ6-SO	36.68 ± 0.65	FS16-SO	—	FS38-SO	33.16 ± 0.06
DD2-SO	—	WQ7-SO	36.55 ± 0.81	FS17-SO	—	FS39-SO	33.77 ± 0.12
JA1-SO	—	WQ8-SO	36.39 ± 0.41	FS18-SO	35.24 ± 0.41	FS40-SO	—
JA2-SO	—	WQ9-SO	34.37 ± 0.74	FS19-SO	—	FS41-SO	—
JA3-SO	—	WQ10-SO	—	FS20-SO	—	FS42-SO	—
JA4-SO	33.89 ± 0.22	WQ11-SO	—	FS21-SO	—	FS43-SO	—
JA5-SO	33.64 ± 0.19	WQ12-SO	—	FS22-SO	36.90 ± 0.24	NG1-SO	—
BS1-SO	—	FS1-SO	35.61 ± 0.33	FS23-SO	—	NG2-SO	—
BS2-SO	—	FS2-SO	36.25 ± 0.59	FS24-SO	—	NG3-SO	36.71 ± 0.42
BS3-SO	—	FS3-SO	35.16 ± 0.73	FS25-SO	—	NG4-SO	—
BS4-SO	—	FS4-SO	—	FS26-SO	37.81 ± 0.08	NG5-SO	—
AT1-SO	—	FS5-SO	32.16 ± 0.51	FS27-SO	36.82 ± 0.22	NG6-SO	37.16 ± 0.19
AT2-SO	—	FS6-SO	32.68 ± 0.19	FS28-SO	36.26 ± 0.51	NG7-SO	—
AT3-SO	38.22 ± 0.36	FS7-SO	—	FS29-SO	—	ddH_2_O	—
AT4-SO	—	FS8-SO	—	FS30-SO	—	Sterile soil	—
AT5-SO	—	FS9-SO	37.13 ± 0.24	FS31-SO	—		
AT6-SO	—	FS10-SO	35.24 ± 0.33	FS32-SO	—		
WQ1-SO	—	FS11-SO	36.49 ± 0.71	FS33-SO	—		

Note: According to LOD validation, a Ct value < 35.45 was considered indicative of *A. panax* detection. The method was first evaluated using artificially inoculated detached leaves, stems, and seeds, and subsequently validated through analysis of natural samples from ginseng-growing areas.

## Data Availability

The original contributions presented in this study are included in the article/Supplementary Material. Further inquiries can be directed to the corresponding authors.

## Supplementary Materials

The following supporting information can be downloaded at: https://www.mdpi.com/article/10.3390/jof12050317/s1, Figure S1: specific primer design. Conventional PCR primers: 1-2F/R; qPCR primers: Q2-1F/R. Table S1: fungal isolates used in this study. Table S2: detailed information on samples. Table S3: Ct values of ten replicates used for the limit-of-detection (LOD) determination.
