# Supplementary material for: A Quantitative Real-Time PCR Assay for Detection and Quantification of the Ginseng Alternaria Leaf and Stem Blight Pathogen Alternaria panax
Source: J Fungi (Basel). 2026 Apr 26;12(5):317. doi: 10.3390/jof12050317 (PMC13208272; doi:10.3390/jof12050317)
Supplement: Supplementary file 1 [file jof-12-00317-s001.zip › Figure S1.pdf]

TCACTTATACTCCCTACTAATCCCCATCTTCAACACCGGTCGCTCCCCAGCAGACTTCTTGCGCTCCCTCGGATACGGTTTGTCGGAC

AGGATAGCGCGTAGATTGGGGTTTGAGACGGCGTGTTTGATAGAGGAGGGGTCGCGGTACGTTGAAGAACGAGGTTTGGGGA

CCGGGAGGGGGCTGGGTGGTTAGTATGTTAGACATGGAAGGATGGAAGGAGAAGGAGAACATACACATACCCCTGACCGGTGA

TGAGGTTGATATGGCTGGGTGTACCACCACCAGCAGCAGTAGACAAAATACTATCAAACAGGCGAATAGGCCTCGGCACCACAGT

AAACGACGTGTCCCGACCGATAGGCGAAATTTGATCCAGCCTGCGCTGAGACTCCGGGAAGACGCGTGTTAATCTGCCACTGAA

CGCAGGCGACGCATGACCTGGGCTCGAGTTGACTTGGCGGATGGGGAGTATGGTGGTGGGATGGGCAGCGGGTGTGGATACG

1-2F

GGTGCTGGGTTGATCGGGGGGAGGATAGGTACCGAACCGGGGGTCGTGGGAGGAGCCATTGTACCACGGAATGCGGCGTCGG

TACGATGTCTGGTAGTACCTTGGGTTCTTGAGCTTGTAGCGCGATCGTTCTGGGGGCGAGTTACGGCAGCGGTAGCGGTTCCGCG

Q2-1F

GACTCGGGGGGTGCGTTGACATCGCTGTGGATGCTTGTCACTGCCACTGCGACTGGTACGACTGGTAGGTGCAGTTGAGCGTCT

GGCACCGATACCCGCGCCAGTGTTTGCGCTAAGGATGTTGCGATCTGCTACAGTTGGGCGTCTAGCGTTGATACCCATATTGGTCG

Q2-1R

GACGAGAAACATCTTCTGCACCACGAGCATGTGCTTGAGGAGTTTCGCGTTGGGTACCGTGATTGCGAGGTGCCAT

1-2R

Figure S1. Specific primer design. Conventional PCR primers: 1-2F/R, amplicon size: 413 bp; qPCR primers: Q2-1F/R, amplicon size: 154 bp, melting point: 86 °C. Species-specific primers for the qPCR assay were developed through the following procedure: First, comparative genomic analysis identified a gene unique to *Alternaria panax* (GenBank ID: KAG9184916.1), encoding a protein with notably low similarity to *A. alternata*, *A. tenuissima*, and *A. brassicicola*, and absent orthologs in *A. solani* and *A. longipes*. Second, primers 1-2F/R targeting a 300–500 bp fragment within this gene were designed for conventional PCR, yielding a 413 bp amplicon. BLASTn analysis revealed no significant identity to non-target species in the NCBI nucleotide database. This fragment was cloned into pUCm-T vector, sequenced, and used as template for subsequent qPCR primer design. Finally, primer pair Q2-1F/R was designed to amplify a 154 bp fragment within the verified region for quantitative PCR, optimizing for real-time detection efficiency and melting curve resolution.
